# Disposable Stainless-Steel Wire-Based Electrochemical Microsensor for In Vivo Continuous Monitoring of Hydrogen Peroxide in Vein of Tomato Leaf

**DOI:** 10.3390/bios12010035

**Published:** 2022-01-12

**Authors:** Doudou Huo, Daodong Li, Songzhi Xu, Yujie Tang, Xueqian Xie, Dayong Li, Fengming Song, Yali Zhang, Aixue Li, Lijun Sun

**Affiliations:** 1 School of Life Sciences, Nantong University, 9 Seyuan Road, Nantong 226019, China; 1909310002@stmail.ntu.edu.cn (D.H.); 2008310015@stmail.ntu.edu.cn (D.L.); xusongzhi@ntu.edu.cn (S.X.); 2009110168@stmail.ntu.edu.cn (Y.T.); 2009110180@stmail.ntu.edu.cn (X.X.); 2 National Key Laboratory for Rice Biology, Institute of Biotechnology, Zhejiang University, Hangzhou 310029, China; dyli@zju.edu.cn (D.L.); fmsong@zju.edu.cn (F.S.); 3 School of Medicine, Nantong University, Qixiu Road 19, Nantong 226001, China; zyl031912@ntu.edu.cn; 4 Beijing Research Center of Intelligent Equipment for Agriculture, Beijing Academy of Agriculture and Forestry Sciences, Beijing 100097, China; liax@nercita.org.cn

**Keywords:** plants, hydrogen peroxide, disposable stainless-steel wire, electrochemical microsensor, in vivo and continuous electrochemical monitoring

## Abstract

As one of the pivotal signal molecules, hydrogen peroxide (H_2_O_2_) has been demonstrated to play important roles in many physiological processes of plants. Continuous monitoring of H_2_O_2_ in vivo could help understand its regulation mechanism more clearly. In this study, a disposable electrochemical microsensor for H_2_O_2_ was developed. This microsensor consists of three parts: low-cost stainless-steel wire with a diameter of 0.1 mm modified by gold nanoparticles (disposable working electrode), an untreated platinum wire with a diameter of 0.1 mm (counter electrode), and an Ag/AgCl wire with a diameter of 0.1 mm (reference electrode), respectively. The microsensor could detect H_2_O_2_ in levels from 10 to 1000 µM and exhibited excellent selectivity. On this basis, the dynamic change in H_2_O_2_ in the vein of tomato leaf under high salinity was continuously monitored in vivo. The results showed that the production of H_2_O_2_ could be induced by high salinity within two hours. This study suggests that the disposable electrochemical microsensor not only suits continuously detecting H_2_O_2_ in microscopic plant tissue in vivo but also reduces the damage to plants. Overall, our strategy will help to pave the foundation for further investigation of the generation, transportation, and elimination mechanism of H_2_O_2_ in plants.

## 1. Introduction

Hydrogen peroxide (H_2_O_2_), a simple molecular compound discovered by Louis Jacques Denard a hundred years ago, is one of the crucial members of the reactive oxygen species (ROS) [1,2]. Compared to the other ROS members such as ^1^O_2_, O_2_, and OH, H_2_O_2_ is more stable. H_2_O_2_ is generally produced in various cellular components such as chloroplast, peroxisomes, and mitochondria, where many metabolic pathways occur in plants [2,3,4,5,6]. The levels of H_2_O_2_ in the fresh leaf extraction were significantly different in different plants under normal growth conditions [7]. For example, the concentrations of H_2_O_2_ ranged from 0.15 μmol·(g·FW)^−1^ in tobacco leaves to 6 μmol·(g·FW)^−1^ in marigold leaves under normal conditions [8,9]. Tewari et al. reported that the localization of H_2_O_2_ in different regions of maize leaf exhibited an increasing pattern from the base to the leaf tip [10]. In general, there is a balance between the production and elimination of H_2_O_2_. However, this balance can be broken by stresses or mechanical wounding [11]. H_2_O_2_ is also a core component of plant signaling networks to regulate plant growth and development, as well as responding to various stresses [1,12]. It has been reported that abscisic acid can induce the production of H_2_O_2_, further inactive Ca^2+^ channels, which are one crucial step for ABA-induced stomatal closing to improve drought resistance [13]. Salicylic acid can elevate the amount of H_2_O_2_ to induce the expression of defense genes to increase the resistance to the pathogen [14,15]. The concentration of H_2_O_2_ can be enhanced when the catalase activity of SABP (salicylic acid bind protein) was inhibited [14,15].

Although studies about the roles of H_2_O_2_ in the signal transduction, especially in crosstalk with the plant hormones in the stresses have made great progress [1,11], direct experimental evidence is lacking regarding the sites, initiation time, and transportation of H_2_O_2_. Obtaining this evidence is limited on how to determine the concentration distribution and dynamic change in H_2_O_2_ in plants. Currently, titration, spectroscopy colorimetry, fluorescence, chemiluminescence, and chromatography are widely used in the detection of the H_2_O_2_ concentration [16,17,18,19,20,21,22,23,24]. The success of these methods is limited by the long time required for sample preparation, especially for separating or grinding plant samples before determination. The complicated and time-consuming pre-treatment of plant samples leads to a loss or change in H_2_O_2_, which makes it difficult to obtain the concentration of H_2_O_2_ in situ and in realtime.

The electrochemical method provides a convenient way for in vivo or real-time detection of IAA, SA, glucose, and sucrose in plants because of its easy operation, high sensitivity, and selectivity for detecting and quantifying [25,26,27]. For detecting the H_2_O_2_ in plants in situ, a few studies such as paper-based electroanalytical devices or microelectrode sensors have been described [28,29,30,31]. In our previous study, a paper-based electroanalytical device provided a rapid and direct electrochemical determination of H_2_O_2_ in tomato leaves inoculated with *Botrytis cinerea*, however, there were still some problems [31]. For example, there was great damage to the plants during the detection of H_2_O_2_. The continuous monitoring of H_2_O_2_ in plants could also not be realized in this system. Single-walled carbon nanotubes and hemoglobin-modified carbon fiber ultramicroelectrodes were used to detect the H_2_O_2_ in vivo on aloe under salinity stress [30]. A platinum disc microelectrode sensor was employed to monitor the H_2_O_2_ in *Agavetequilana* leaves after inoculation with endophytic bacteria [29]. Although these microelectrode sensors could be used to detect H_2_O_2_ [28,29,30], microelectrode sensors that can be prepared simply and are low-cost and disposable are still needed. Recently, stainless-steel materials have been used as electrodes to detect dopamine, levodopa, uric acid, and heavy metals due to their excellent properties such as good electrical conductivity, lowcost, high mechanical strength, environmentally friendly, and commercial availability [32,33,34,35]. More importantly, the diameter of stainless-steel wires can be reduced to the micrometer level, which is very suited to fabricate the microelectrode sensors. For example, 0.127 mm stainless-steel wires were used as the electrodes in the biobotic [36].

In this study, a disposable microsensor based on 0.1 mm stainless-steel wire was constructed for electrochemical detection of H_2_O_2_. This microsensor showed excellent electrochemical detection performance. The dynamic change in H_2_O_2_ in the vein of tomato leaf under high salinity was continuously monitored in vivo. This study suggested that the disposable electrochemical microsensor is not only suitable for continuously detecting H_2_O_2_ in microscopic plant tissue in vivo but can also reduce the damage to plants, which will help pave the foundation for further investigation of the generation, transportation, and elimination mechanisms of H_2_O_2_ in plants.

## 2. Materials and Methods

### 2.1. Chemicals and Materials

Hydrogen tetrachloroaurate (III) trihydrate (HAuCl_4_·3H_2_O) was obtained from Sigma (St. Louis, MO, USA). The 304 stainless-steel wire (SS, d = 0.1 mm), platinum wire (Pt, d = 0.1 mm), and silver wire (Ag, d = 0.1 mm) were obtained from Alfa Aesar Chemical Co., Ltd. (Shanghai, China). The sample capillary was obtained from Shanghai Dongming Glass instrument Co., Ltd. (Shanghai, China). Double-conductor copper foil tape was obtained by Shenzhen Wangxing Tape Co., Ltd. (Shenzhen, China). The 3M superglue was obtained from 3M China Co., Ltd. (Shenzhen, China). The tomato seeds of one generation hybrid, Shanghai906, were obtained from Lintong Changfeng Vegetable Breeding Farm (Xi’an, China). H_2_O_2_ was purchased from Shanghai Zhanyun Chemical Co., Ltd. (Shanghai, China). All the other chemical reagents were analytical grade. Double distilled water was used in all experiments.

### 2.2. Material Preparation

HAuCl_4_·3H_2_O (10 g/L) was distilled by the double distilled water to prepare 0.5 mmol/L HAuCl_4_·3H_2_O for electroplating. Thirty percent of H_2_O_2_ was diluted to different concentrations by 0.2 M phosphate-buffered solution (PBS) with pH 7.0 for detection. Tomato plants were grown in a mixture of perlite: plant ash: vermiculite (1:2:6) in a growth room at 22 °C. The lighting regime for the tomato plants was 16 h of light/8 h dark at a total light intensity of around 540 μmol/m^2^/S. For analysis of the production and transportation of H_2_O_2_ in the vein of tomato leaf response to high salinity stress, six-week-old tomato was treated with 0.4 M NaCl solution. As shown in Figure S1, the lateral stem of tomato was cut and the end of the stem was wrapped with cotton soaked in water (control) or 0.4 M NaCl solution (high salinity stress).

### 2.3. The Preparation of SS Wire-Based Electrochemical Microsensor

**Scheme 1:**
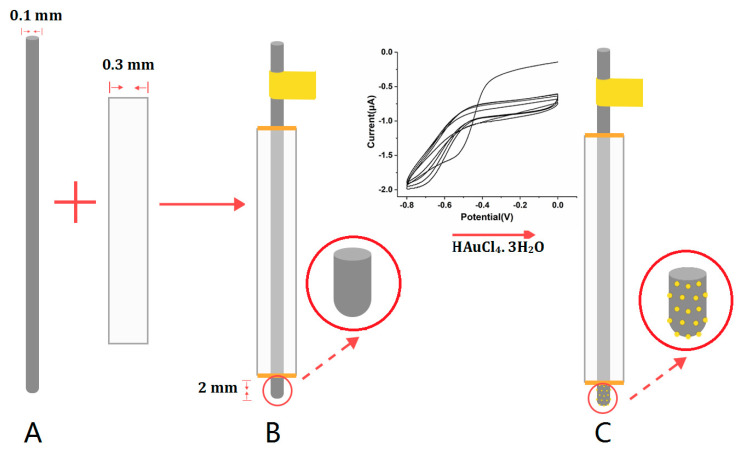
The preparation process of disposable stainless-steel (SS) wire-based electrochemical microsensor system: (**A**) bare SS electrode; (**B**) fixed-bare SS electrode in capillary; (**C**) the nano-Au modified bare SS electrode (Au/SS).

The SS wire, Pt wire, and silver wire were cut into 50 mm segments and washed with ethanol, water under ultrasonic for 10 min, and dried at room temperature, respectively. The capillaries (d = 0.3 mm) were cut into 35 mm segments and an SS wire was placed into the capillary (Scheme 1A). One end of the SS wire was exposed to a length of 2 mm for sample testing, and the other end of the SS wire was exposed to a length of 13 mm for linking to the electrochemical workstation. The SS wire was fixed in the capillary by the 3M super glue. Finally, copper conductive tape was applied to the end of the 13 mm SS wire (Scheme 1B). Then, electrodeposition of nano-Au on the bare SS was carried out in a 0.5 mmol/L HAuCl_4_ solution under the potential from −0.8 to 0 V by cyclic voltammetry (CV) (Scheme 1C). The modified SS wire was used as a working electrode (Au/SS electrode). The unmodified Pt wire (counter electrode) was fixed in the capillary in the same way as the SS wire. For the reference electrode, the 0.1 mm silver wire was fixed in the capillary in the same way as the Pt electrode. Then, the fixed-silver wire was soaked in 84 disinfectant (the main composition is NaClO) for 30 min to generate a silver/silver chloride electrode (Ag/AgCl).

### 2.4. Electrochemical Measurements

All electrochemical experiments were carried out on a CHI 1240C electrochemical station. The electrocatalytic behaviors of the electrochemical detection system toward H_2_O_2_ were investigated by CV with the potential from −1.0 to 0.6 V. H_2_O_2_ was detected by an amperometric i-t curve with an initial potential of −0.6 V, sample interval of 0.1 s, running time of 1500 s, quiet time of 0 s, scales during a run of 1 on the SS wire-based electrochemical microsensor.

### 2.5. qRT-PCR Analysis of Gene Expression

In order to evaluate the level of H_2_O_2_ in the vein of tomato leaves, the expression of *SLRbohA* and *SLRbohB* was analyzed. Total RNA was extracted from the vein of tomato leaves (0.20 g fresh weight) with TRIzol (Invitrogen, Shanghai, China) and then treated with RNase-free DNase (TaKaRa, Dalian, China). First-strand cDNAs were synthesized by AMV reserve transcriptase (TaKaRa, Dalian, China) as instructed. The obtained cDNA was used for gene expression analysis with qRT-PCR. Each qPCR reaction contained 0.1 μg cDNA, 7.5 pmol of each gene-specific primer (Table S1), and 12.5 μL × 2 SYBR premix Ex TaqTM (TaKaRa, Dalian, China) in a final volume of 25 μL. The qRT-PCR was performed in a Roche *LC480* real-time quantitative polymerase chain reaction detection system. The relative gene expression levels were calculated using the 2^−ΔΔCT^ method as described [37]. Three independent biological replicates were analyzed.

## 3. Results and Discussion

### 3.1. Characterization of theModified Electrodes

**Figure 1:**
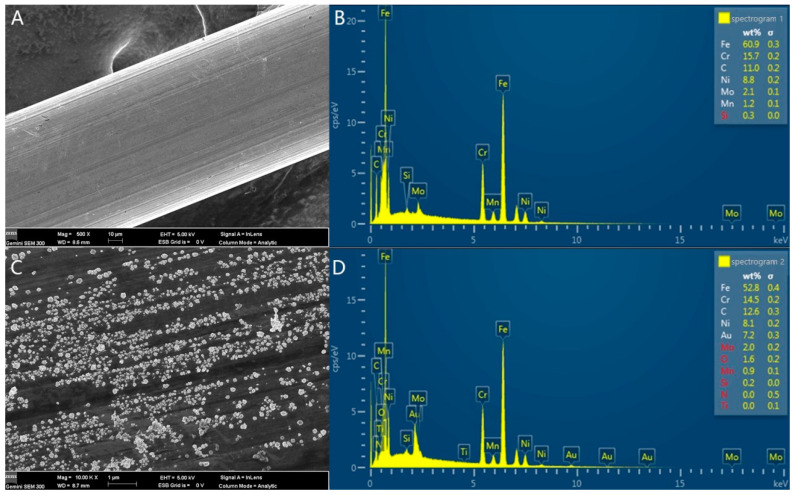
The SEM (**A**,**C**) and EDS (**B**,**D**) spectrum analysis of bare SS and Au/SS electrode.

The surface characterization of the electrode was observed using a scanning electron microscope (ZEISSSEM300, Oberkochen, Germany). As shown in Figure 1, the surface of the bare SS wire electrode with a diameter of 0.1 mm is smooth (Figure 1A).The electrodeposition methods were used to deposit gold nanoparticles on the bare SS wire electrode. Compared to the bare SS electrode, the 5~15 nm gold nanoparticles homogeneously distributed throughout the surface of the bare SS wire electrode (Figure 1C). The element composition of the modified electrodes was also characterized by an energy dispersive spectrometer (Oxford Instruments, UK). As shown in Figure 1B, the main element of bare SS wire electrode was Fe, above 60.9%. After electrodepositing of Au, the elemental composition of Fe decreased to 52.8%, while the Au increased from 0% to 7.2% (Figure 1D). All of these results suggested the gold nanoparticles were electroplated on the bare SS wire electrode.

### 3.2. The Electrochemical Performance of the Au/SSWire-Based Electrochemical Microsensor

**Figure 2:**
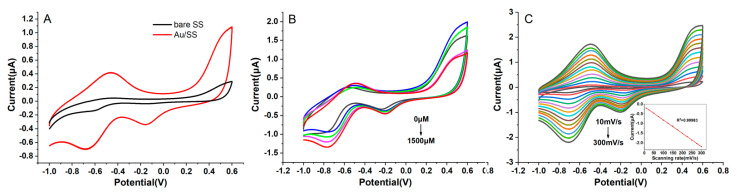
(**A**) CV curves of bare SS, and Au/SS wire-based electrochemical microsensor in the presence of 100 μM H_2_O_2_ in 0.2M PBS with pH 7 solution at the scanning rate of 100 mV·s^−1^. (**B**) CV curves of Au/SS wire-based electrochemical microsensor in the absence of H_2_O_2_ and the presence of different concentrations of H_2_O_2_ in 0.2M PBS with pH 7 solution at the scanning rate of 100 mV·s^−1^. The concentrations of H_2_O_2_ were 0 μM (control, black line), 100 μM (blue line), 500 μM (green line), 1000 μM (pink line), and 1500 μM (red line), respectively. (**C**) CV curves of SS wire-based electrochemical microsensor at different scanning rates in 100 μM H_2_O_2_ solution. The scanning rate ranges from 10 to 300 mV·s^−1^.

The electrocatalytic behavior of the Au/SS wire-based electrochemical microsensortoward H_2_O_2_ reduction was investigated using CV. There was only one reduction peak of CV curves at around −0.7 V on the bare SS wire electrode in the presence of 100 μM H_2_O_2_ (Figure 2A).Compared to the bare SS wire electrode, the CV curves changed with an obvious reduction peak at around −0.7 V and −0.2 V on the Au/SS electrode (Figure 2A). Such results showed that Au nanoparticles possess exceptional electrical conductivity and electrocatalytic activity, which could improve the electrochemical response of H_2_O_2_. Moreover, with the addition of H_2_O_2_ from concentrations of 0 to 1500 μM, the reduction current of H_2_O_2_ at around −0.7 V was enhanced gradually, while there was no obvious increase at −0.2 V, which suggested that the electrocatalytic reaction of H_2_O_2_ mainly occurs at −0.7 V (Figure 2B). Figure 2C shows the curves of the SS wire-based electrochemical microsensor system with the increase in scanning rates from 10 to 300 mV/s. The results showed that the reduction peak current of 100 μM H_2_O_2_ decreased linearly with the increase in the scan rates, which suggested that the redox process of the modified electrode surface is an adsorption-controlled process with an irreversible electroreduction of H_2_O_2_. In addition, the reduction peak potential of H_2_O_2_ at around −0.7 V gradually moves toward the negative direction with the increase in scanning rates, which is possibly caused by the torpid electron transfer kinetics.

### 3.3. Amperometric Detection of H_2_O_2_ with the Au/SS Wire-Based Electrochemical Microsensor

**Figure 3:**
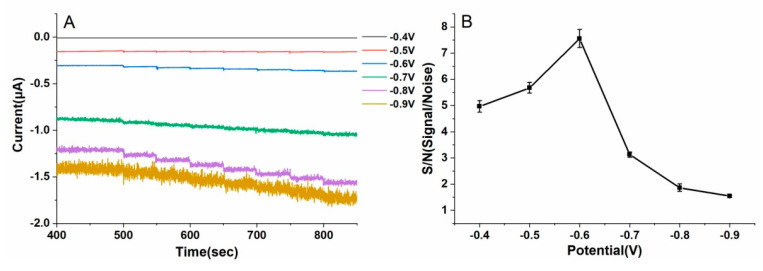
(**A**) The amperometric response of Au/SS wire-based electrochemical microsensor to continuous additions 10 µLH_2_O_2_ with concentration 100 mMin 10 mL solution with 0.2 M PBS (pH 7) at different application potentials. (**B**) The relationship between S/N ratio and applied potential (*n* = 7).

Considering the great influence of potential on the sensitivity, the amperometric i-t method was used to estimate the influence of the applied potential on the Au/SS wire-based electrochemical microsensor. As shown in Figure 3A, the influence of different applied potentials (−0.9, −0.8, −0.7, −0.6, −0.5, and −0.4 V) on the amperometric response of the Au/SS wire-based electrochemical microsensor system was investigated by continuously adding 100 µM H_2_O_2_ into 0.2 M PBS solution. The signal-to-noise ratio (a ratio of the current response signal to background noise, S/N ratio) can reflect the sensitivity of the detection system. It was observed that the S/N ratio increased and reached a maximum value at −0.6 V, then gradually decreased (Figure 3B). Therefore, an applied potential of −0.6 V for the amperometric i-t method was selected in the following experiments. Whether the untreated stainless-steel wires could be used as reference electrodes and counter electrodes was studied. Compared to the present electrode system, the untreated stainless-steel wire could not be used as reference electrode and counter electrodes due to the big background noise (Figure S2). The influence of working electrodes fabrication with the different steel wire diameters (0.1, 0.05, and 0.01 mm) on the electrochemical detection of H_2_O_2_ was also studied. The results showed that the highest current response was the steel wire with a diameter of 0.1 mm (Figure S3).

**Figure 4:**
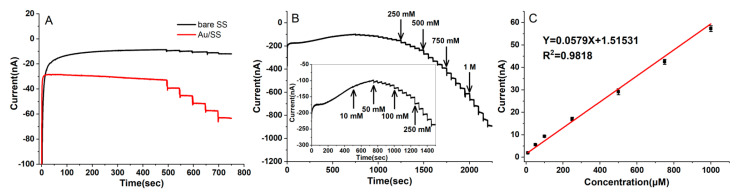
(**A**) Amperometric response of the bare SS and Au/SS wire-based electrochemical microsensor to successive additions of 10 µL H_2_O_2_ with concentration100 mM in 10 mL solution with 0.2 M PBS (pH 7) at −0.6 V application potential. (**B**) Amperometric response of the Au/SS wire-based electrochemical microsensor to successive additions of 10 µL H_2_O_2_ with concentration 10 mM, 50 mM, 100 mM, 250 mM, 500 mM, 750 mM, and 1000 mM, respectively, in 10 mL solution with 0.2 M PBS (pH 7) at −0.6 V application potential. (**C**) Linear relation between the amperometric response and H_2_O_2_ concentration ranging from 10 µM to 1000 µM (*n* = 5).

**Figure 5:**
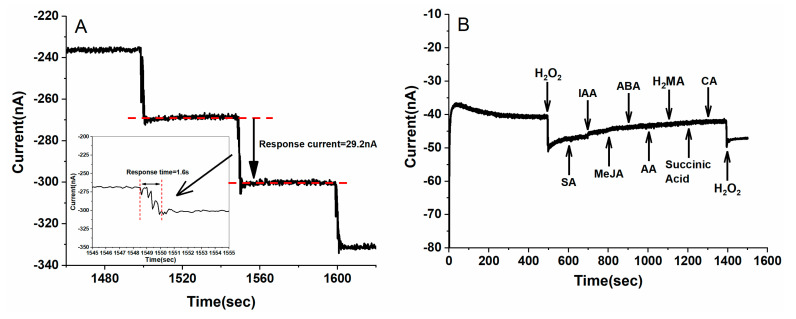
(**A**) Response time of the Au/SS wire-based electrochemical microsensor to the H_2_O_2_. Amperometric response to continuous additions of 10 µL H_2_O_2_ with concentration 500 mM in 10 mL solution with 0.2 M PBS (pH 7) at −0.6 V application potential. (**B**) The ant-interference ability of the SS wire-based electrochemical microsensor for detection of H_2_O_2_. Amperometric response to continuous additions of 10 µL H_2_O_2_, salicylic acid (SA), indole-3-acetic acid (IAA), methyl jasmonate (MeJA), abscisic acid (ABA), ascorbic acid (AA), malic acid (H_2_MA), succinic acid, and citric acid (CA), and H_2_O_2_ with concentration 100 mM, respectively, in 10 mL solution with 0.2 M PBS (pH 7) at −0.6 V application potential.

The typical amperometric (i-t) curves of the Au/SS wire-based electrochemical microsensor were recorded by the successive addition of H_2_O_2_ with different concentrations into 0.2 M PBS solution at an applied potential of −0.6 V (Figure 4). The response of the H_2_O_2_ on the bare SS and Au/SS wire electrodes was also compared. As shown in Figure 4A, the response of H_2_O_2_ on the Au/SS wire electrode was higher than the SS wire, which showed that Au nanoparticles modified SS wire could improve electrochemical detection of H_2_O_2_.The current response value increased with the increase in H_2_O_2_ concentration (Figure 4B). There was a good linear relationship between the current variation of H_2_O_2_ and the concentration of H_2_O_2_ in the range of 10 μM to 1000 μM (Figure 4C). After adding 500 μM H_2_O_2_ in stirring PBS solution, the reduction current of the Au/SS wire-based electrochemical microsensor system showed a significant increase and obtained the maximum steady-state current within 1.6 s (Figure 5A). The possible interference from the plant molecules in plant fluids for the determination of H_2_O_2_ in plants was also investigated. Figure 5B shows that no significant interferences were observed for the determination of H_2_O_2_ in the presence of salicylic acid (SA), indole-3-acetic acid (IAA), methyl jasmonate (MeJA), abscisic acid (ABA), ascorbic acid (AA), malic acid (H_2_MA), succinic acid, and citric acid (CA) with 100 μM, respectively. These results suggested that the Au/SS wire-based electrochemical microsensor system can be used for the determination of H_2_O_2_ in real samples. The reproducibility of the Au/SS wire-based electrochemical microsensor was evaluated from the response on 100 μM H_2_O_2_ using six working electrodes. The relative standard deviation was about 5.8% and indicated a good reproducibility of the Au/SS wire-based electrochemical microsensor. The limit of detection (LOD) was 3.97 μM. The stability of the Au/SS wire-based electrochemical microsensor was also tested. The results showed that the Au/SS wire-based electrochemical microsensor could be used at least seven times and maintained stability for at least seven days (Figure S4). These results suggested that the Au/SS wire-based electrochemical microsensor system can be used for the determination of H_2_O_2_ in real samples. Compared with previous reports [28,29,30,31,38,39,40], the disposable SS wire-based electrochemical microsensor was superior regarding the low cost (about USD 0.02, suitable for mass production), simple preparation (less than 20 min to prepare this system), good analytical performance, and in vivo and continuous electrochemical monitoring of H_2_O_2_ in plants (Table S2).

### 3.4. In Vivo and Continuous Electrochemical Monitoring of H_2_O_2_ in Vein of Tomato Leaf

**Figure 6:**
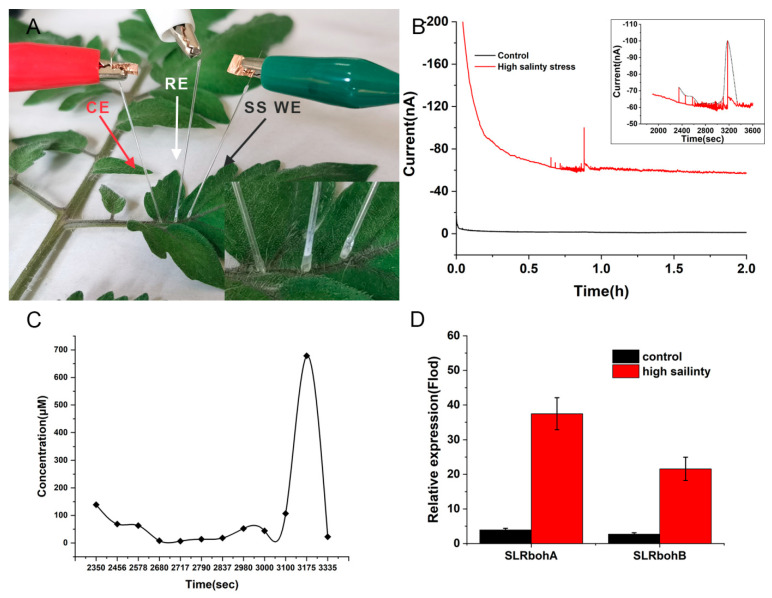
(**A**) In vivo and continuous electrochemical monitoring of H_2_O_2_ in vein of the tomato leave based on the Au/SS wire-based electrochemical microsensor, and the inset showed the enlarged part of the analysis area. (**B**) The typical amperometric i-t detection curve of H_2_O_2_ in vein of the tomato leaves under the high salinity stress, and the inset showed the enlarged part of H_2_O_2_ from 40 to 60 min. (**C**) illustrated the dynamic change in H_2_O_2_ in vein of tomato leaf. (**C**) The dynamic change in H_2_O_2_ in vein of tomato leaf under the high salinity stress. (**D**)The expression level of *SLRbohA* and *SLRbohB* under the high salinity stress.

To study the production of H_2_O_2_ in the veins of the tomato leaf under high salinity stress, H_2_O_2_ was continuously monitored in vivo and based on the Au/SS wire-based electrochemical microsensor. As shown in Figure 6A, the working electrode, counter electrode, and reference electrode of the Au/SS wire-based electrochemical microsensor were placed into the vein of the tomato leaf, followed by transferring 5 μL 0.2 M PBS (pH 7) on the surface of electrodes for electrochemical detection. The typical amperometric i-t detection curves of H_2_O_2_ in the vein of tomato with or without high salinity stress for 2 h are shown in Figure 6B. The results showed that the records of control remained flat during the continuous measurement which suggested a stable H_2_O_2_ concentration in the vein of tomato leaf. With the high salinity stress, H_2_O_2_ was observed to increase about 40 min and resulted in a large peak at 60 min (Figure 6B). Figure 6C illustrates the dynamic change in H_2_O_2_ in the vein of tomato leaf from 6.7 to 678.6 μM. In vivo H_2_O_2_ monitoring with high salinity stress was repeated three times and each experiment yielded similar results of excess H_2_O_2_ accumulation (Figure S5). Respiratory burst oxidase homologs (Rbohs) also known as NADPH oxidases, are key enzymes that can catalyze the generation of ROS in plants [41]. Rbohs-generated ROS has been shown to regulate responses to many abiotic stresses such as drought, heat, cold, and salinity [42,43]. Stresses are shown to alter the levels of different plant hormones that in turn regulate RBOHs activity and ROS production via many different mechanisms [43]. Eight *SlRboh* genes (*SlRbohA~SlRbohH*) were identified in tomato [44]. Among them, *SLRbohB* has been proven to positively regulate resistance to *B. cinerea* and drought stress tolerance in tomato [44]. Therefore, the expression analysis of *SlRbohA* and *SlRbohB* in our studies was performed to confirm our detection results. Comparing the control treatments, the expression levels of *SlRbohA* and *SlRbohB* in the veins of tomato leaves under high salinity treatment showed a >8-fold increase at 1 h post treatment, which related to the increase in H_2_O_2_ in the vein of tomato under high salinity stress (Figure 6D). These results suggested that our approach might provide an effective way for in vivo and continuous detection of H_2_O_2_ in plants.

## 4. Conclusions

In this study, we developed a disposable Au/SS wire-based electrochemical microsensor to detect H_2_O_2_ in vivo and continuously. Compared to the traditional methods, the complicated and time-consuming treatment processing for quantification of H_2_O_2_ in the plant samples was avoided, which could ensure continuously obtaining the H_2_O_2_ concentration in vivo. With our approach, the dynamic change in H_2_O_2_ in the vein of tomato leaf under high salinity was continuously monitored in vivo. Our strategy will help to pave the foundation for further investigation of the generation, transportation, and elimination mechanisms of H_2_O_2_ in plants.

## Data Availability

Not applicable.

## Supplementary Materials

The following are available online at https://www.mdpi.com/article/10.3390/bios12010035/s1, Table S1: The qRT-PCR primers; Table S2: List of analytical performance of electrochemical methods for detection of H_2_O_2_ in the plants; Figure S1: The method of high salinity stress for the tomato; Figure S2: (A) Pt electrode was used as counter electrode and Ag/AgCl electrode as reference electrode. (B) SS electrodes were used as counter electrode and reference electrode. The Amperometric response of the Au/SS wire-based electrochemical microsensor to successive additions of 10 µL H_2_O_2_ with concentration 100 mM in 10 mL solution with 0.2 M PBS (pH 7) at −0.6 V application potential; Figure S3: (A) The Amperometric response of the Au/SS wire with the different diameter based electrochemical microsensor to successive additions of 10 µL H_2_O_2_ with concentration 100 mM in 10 mL solution with 0.2 M PBS (pH 7) at −0.6 V application potential. (B) The relationship between the amperometric response and the diameter of SS (*n* = 5); Figure S4: (A) One Au/SS wire-based electrochemical microsensor was repeated for 7 times.(B) Seven Au/SS wire-based electrochemical microsensors were fabricated at the same time and then were tested at following seven days. Amperometric response of the Au/SS wire-based electrochemical microsensor to successive additions of 10 µL H_2_O_2_ with concentration 100 mM in 10 mL solution with 0.2 M PBS (pH 7) at −0.6 V application potential; Figure S5: The in vivo H_2_O_2_ monitoring in vein of tomato with high salinity stress.
