# Supplementary material for: Disposable Stainless-Steel Wire-Based Electrochemical Microsensor for In Vivo Continuous Monitoring of Hydrogen Peroxide in Vein of Tomato Leaf
Source: Biosensors (Basel). 2022 Jan 12;12(1):35. doi: 10.3390/bios12010035 (PMC8773776; doi:10.3390/bios12010035)
Supplement: Supplementary file 1 [file biosensors-12-00035-s001.zip › biosensors-1522888-supplementary.pdf]

biosensors

MDPI

Supplementary Materials: Disposable Stainless-Steel Wire-Based Electrochemical Microsensor for In Vivo Continuous Monitoring of Hydrogen Peroxide in Vein of Tomato Leaf

Doudou Huo[1,†], Daodong Li[1,†], Songzhi Xu[1,†], Yujie Tang[1], XueqianXie[1], Dayong Li[2], Fengming Song[2], Yali Zhang[3], Aixue Li[4] and Lijun Sun[1,*]

Table S1. The qRT-PCR primers.

Gene Name	Primer Set	
	Forward Primer (5'–3')	Reverse Primer (5'–3')
SLRbohA	GAGAGTAGGATTCAGCGGT	GCCTCTTTTCGAGCTTGCT
SLRbohB	AGGGAATGATAGAGCGTCG	CATCGTCATTGGACTTGGC
β-actin	GTCCTCTTCCAGCCATCCAT	ACCACTGAGCACAATGTTACCG

Table S2. List of analytical performance of electrochemical methods for detection of H_2O_2 in the plants.

Electrode System	Price of Working Electrode	Method	Applied Potential	Liner Range	The Response Time	Sample Application	The Time of Electrode Preparation	Ref
WE: Au nanodots-ITO CE: Platinum wire RE: Ag/AgCl wire	0.5 $	Differential pulse voltammetry	−0.90V	0~1000 μM	No description	Detection of the direct tomato leaves (3mg)	5 min	25
WE: HRP/Cys/AuNPs/ITO CE: platinum foil RE: Saturated calomel electrode	1 $	Amperometric	−0.15 V	8~ 3000 μM	Less than 5 s	Detection of the extraction solution of the plant leaves	More than 12 hours	29
WE: Hb/SWCNTs/CFUMEs(8 μm) CE: Pt wire（0.1 mm） RE: Ag/AgCl $_{(sat.\ kCl)}$ (1 mm)	1 $	Amperometric	−0.1V	4.9~405 μM	No description	Detection of aloe leaves In vivo and continuous	More than 1 hour	24
WE: HRP/colloidal Au/ pPA/Pt electrode (1 mm) CE: Pt wire RE: Saturated calomel electrode	10 $	Amperometric	0 V	0.42~1500 μM	Within 5 s	Detection of the extraction solution of the plant leaves	More than 18 hours	27
WE: Platinum disc microelectrode(0.050 mm) RE: Silver expoxy coat, Ag/AgCl $_{(sat.\ kCl)}$	10 $	Cyclic voltammogram	−1.0~0V (−0.7V)	0.1mM~100 mM	No description	Detection of agave tequilana leaves In vivo	20 min	23
WE：Au nanodots /SS electrode (0.1 mm) CE: Pt wire (0.1 mm) RE: Ag/AgCl wire (0.1 mm)	0.02 $	Amperometric	−0.2 V	10~1000 μM	2.3 s	Detection of the tomato vein In vivo and continuous	20 min	This study

Note: WE, Work electrode; CE, Counter electrode; RE, Reference electrode; Au nanodots-ITO, nano-gold modified indium tin oxide electrode; HRP/Cys/AuNPs/ITO, Horseradish peroxidase/L-Cysteine/gold nanoparticles /indium tin oxide electrode; Hb/SWCNTs/CFUMEs, Hemoglobin/ Single-walled carbon nanotubes/ Carbon fiber ultramicroelectrode; HRP/colloidal Au/ pPA/Pt electrode, Horseradish peroxidase/colloidal Au/poly 2,6-pyridinediamine/platinum wire electrode, Au nanodots /SS, nano-gold modified stainless steel wire electrode.

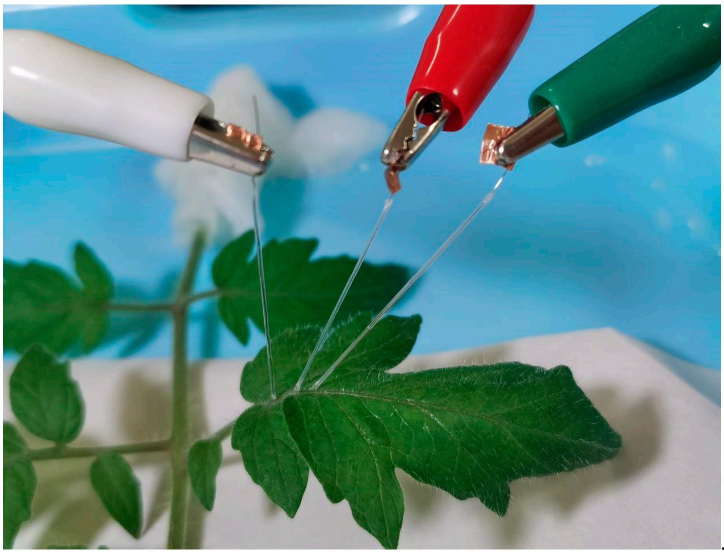

Figure S1. The method of high salinity stress for the tomato.

The lateral stem of tomato was cut and the end of the stems was wrapped with cotton soaked in water (control) or high salinity stress.

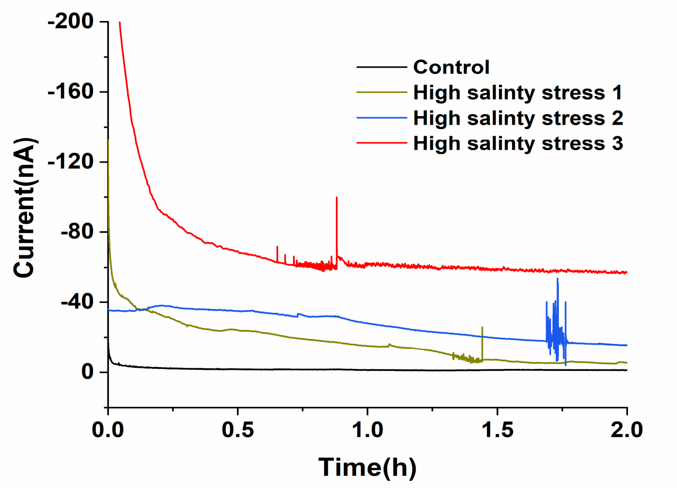

Figure S2. In vivo H_2O_2 monitoring with stress repeated 3 times.
